# Refined tamoxifen administration in mice by encouraging voluntary consumption of palatable formulations

**DOI:** 10.1038/s41684-024-01409-z

**Published:** 2024-07-30

**Authors:** Dominique Vanhecke, Viola Bugada, Regula Steiner, Bojan Polić, Thorsten Buch

**Affiliations:** 1 Institute of Laboratory Animal Science, University of Zurich, Zurich, Switzerland; 2 Institute of Clinical Chemistry, University and University Hospital of Zurich, Zurich, Switzerland; 3 Department of Histology and Embryology, Faculty of Medicine, University of Rijeka, Rijeka, Croatia

**Keywords:** Transgenic organisms, Ethics, Animal behaviour, Gene expression, Assay systems

## Abstract

Drug administration in preclinical rodent models is essential for research and the development of novel therapies. Compassionate administration methods have been developed, but these are mostly incompatible with water-insoluble drugs such as tamoxifen or do not allow for precise timing or dosing of the drugs. For more than two decades, tamoxifen has been administered by oral gavage or injection to CreER^T2^–*loxP* gene-modified mouse models to spatiotemporally control gene expression, with the numbers of such inducible models steadily increasing in recent years. Animal-friendly procedures for accurately administering tamoxifen or other water-insoluble drugs would, therefore, have an important impact on animal welfare. On the basis of a previously published micropipette feeding protocol, we developed palatable formulations to encourage voluntary consumption of tamoxifen. We evaluated the acceptance of the new formulations by mice during training and treatment and assessed the efficacy of tamoxifen-mediated induction of CreER^T2^–*loxP*-dependent reporter genes. Both sweetened milk and syrup-based formulations encouraged mice to consume tamoxifen voluntarily, but only sweetened milk formulations were statistically noninferior to oral gavage or intraperitoneal injections in inducing CreER^T2^-mediated gene expression. Serum concentrations of tamoxifen metabolites, quantified using an in-house-developed cell assay, confirmed the lower efficacy of syrup- as compared to sweetened milk-based formulations. We found dosing with a micropipette to be more accurate than oral gavage or injection, with the added advantage that the method requires little training for the experimenter. The new palatable solutions encourage voluntary consumption of tamoxifen without loss of efficacy compared to oral gavage or injections and thus represent a refined administration method.

## Abstract (web summary)

Pairing palatable formulations with micropipette dosing for administration of water-insoluble drugs such as tamoxifen in mice promotes voluntary consumption without loss of efficacy compared to more invasive administration methods.

## Main

The increased efforts to conduct more humane animal research (3R principle^1,2^) include the development and application of animal-friendly drug administration methods. Unlike water-soluble drugs, most water-insoluble drugs cannot be mixed with drinking water or chow. Instead, they are typically administered via oral gavage (OG) or intraperitoneal (IP) or subcutaneous injection. These invasive interventions can induce stress-related responses in rodents, as reflected by increased stress hormone levels or heart rates^3–8^. Furthermore, repeated OG can increase the risk of unintentional injuries, including perforation of the trachea, esophagus or stomach, introduction of fluids into the trachea or lung, and hemorrhage^9^. Repeated IP injections have been reported to cause local irritation, pain, infection and damage to surrounding tissue^10^.

Recently, a procedure for drug administration in mice that aims to minimize the above-mentioned disadvantages has been proposed as an alternative to OG or IP injections^7,8^. This so-called micropipette-guided drug administration makes use of a sweetened condensed milk solution as a vehicle to motivate mice to voluntarily consume drug solutions. This noninvasive procedure was shown to achieve pharmacokinetic profiles similar to those obtained by OG^7^ or IP injections^8^. However, until now, similar pipette feeding has not been successfully adopted for the administration of water-insoluble compounds such as tamoxifen (TAM).

TAM is a selective estrogen receptor (ER) modulator that is widely used in clinical and basic research applications. For more than 20 years, TAM has been utilized in research to induce spatiotemporal modifications in gene expression in CreER^T1/T2^–*loxP* transgenic mouse models^11–14^. The number of different CreER^T1^ and CreER^T2^ mouse strains generated for research exceeded 1,400 by May 2023, of which more than 200 new strains were generated since May 2021 (refs. ^15–17^). Despite its routine use in research, there is no consensus on the best method for TAM delivery^11^. In most cases, TAM is administered via OG or IP injections but also occasionally by subcutaneous injections or medicated diets^18,19^. While injections allow controlled dosing and timed treatments, the oil- or ethanol-based vehicles required to dissolve TAM can cause local adverse reactions at injection sites^10,11,20^. Oral administration is more physiologically relevant for TAM because TAM first needs to be metabolized by the liver into the biologically active metabolites 4-hydroxytamoxifen and endoxifen^21,22^. However, of the existing oral administration methods, OG is an invasive method that requires restraint of the animal and specific training by the experimenter. Treatment with TAM-supplemented diets, on the other hand, while convenient, does not allow accurate dosing and can result in adverse effects from poor feeding due to aversion^23^.

Developing a more refined method for TAM administration would not only benefit the well-being of experimental mice, but it should also improve experimental outcomes. Indeed, prevention of possible stress-related confounder effects^24–27^ and increased accuracy of dosing and timing of TAM treatments are expected to improve experimental conditions and, thus, the quality of the results. This, in turn, will reduce overall animal use since smaller treatment groups are required to yield statistically significant results. A refined administration method could also benefit research involving other water-insoluble drugs such as cyclosporin A or antibiotics, including linezolid and vancomycin, which are currently administered to mice or rats by OG or IP injections^28–30^. Developing palatable formulations for animal-friendly administration of water-insoluble drugs could even have a larger impact, considering that approximately 40% of drugs with market approval and nearly 90% of molecules in the discovery pipeline are poorly water soluble^31^.

In this study, we developed sweetened formulations that are compatible with water-insoluble drugs such as TAM and that encourage mice to voluntary consume the drug while retaining efficient TAM-mediated induction of gene expression. Our results show that formulations in which TAM is first dissolved in oil and then dispersed in sweetened milk or syrup encourage mice to voluntarily consume TAM offered with a micropipette. The efficacy of the new formulations, as reflected by TAM-mediated genetic recombination, was tested in two CreER^T2^-based transgenic mouse models. First-pass metabolism was assessed by comparing serum concentrations of TAM metabolites from treated mice using a cell-based in vitro assay. Together, these results demonstrate that feeding TAM–sweetened-milk formulations is a more animal-friendly administration method to replace OG and IP injections and, therefore, could become the method of choice when administering water-insoluble drugs.

## Results

### Mixing oil with sweetened solutions makes it palatable to mice

**Fig. 1:**
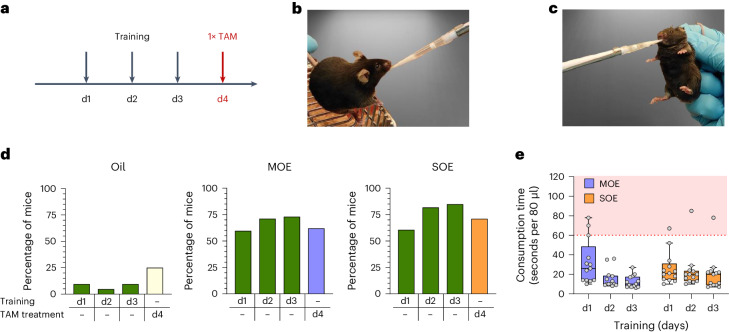
Sweetened oil emulsions and training improve voluntary drug consumption. **a**, Adult male and female mice were trained for 3 days to consume 80 μl of formulations from a disposable plastic micropipette tip and on the fourth day were offered the same formulation containing TAM. **b**, Feeding time was recorded and considered voluntary if the mouse, held only slightly by the proximal tail section, drank the offered volume in less than 60 s. **c**, Any formulation not consumed after 60 s was offered again after gently restraining the mice by the scruff, and the extra time required to drink the remaining solution was recorded. **d**, The fraction of mice that voluntarily consumed (<60 s) oil (*n* = 21), sweetened MOE (*n* = 52) or SOE (*n* = 33) during 3 days of training (green bars) or TAM-containing formulations on the fourth day. Yellow bar, OIL-TAM (*n* = 12); blue bar, MOE-TAM (*n* = 17); orange bar, SOE-TAM (*n* = 17). The oil used for the formulations in **d** was sterilized by heat treatment. **e**, Total consumption time for three training days, recorded from the moment the MOE or SOE formulation was offered (*n* = 13 per group). Indicated is the time before restraining (white zone) as described in **b** and after restraining (red zone) as described in **c**. The oil used for the emulsions in **e** was sterilized by filtration. Minimum and maximum values, interquartile range and median are depicted as Tukey box plots with individual data points shown as gray circles. Source data

To adapt the micropipette-guided drug administration to TAM, we first compared the palatability of TAM dissolved in oil before and after adding sweetened condensed milk or berry syrup. To encourage voluntary consumption and drinking from a micropipette tip^7,8,10^, adult mice were trained for 3 days with the different formulations before being offered the same formulation containing TAM on the fourth day (Fig. 1a). The palatability of the solutions was assessed by recording the time the mice needed to consume a specific volume^32^. We defined consumption as voluntary when mice drank the substance within 60 s while sitting on the cage grid. To prevent the mice from wandering or leaving the grid, they were gently held by the tail (Fig. 1b, Supplementary Video 1 and detailed in Methods). When offered peanut oil (Fig. 1d, first panel) or corn oil (not shown), vehicles that are typically used to dissolve and administer TAM via OG, almost none of the mice voluntarily drank the oil during training or when subsequently offered oil-containing TAM.

The addition of sweetened milk has been shown to encourage mice to voluntarily consume water-soluble drugs^7,8^. However, simply mixing oil with sweetened condensed milk to improve palatability generated solutions that quickly coalesced (data not shown), regardless of how long the solutions were mixed with an electric vortex mixer. By contrast, using a simple high-energy homogenization method, the so-called two-syringe method^33^ (Supplementary Fig. 1a and described in Methods), resulted in stable oil-in-water emulsions. We retained two promising formulations: a milk–oil emulsion (MOE) made with sweetened condensed milk and a syrup–oil emulsion (SOE) made with berry syrup. The mean diameter and standard deviation (s.d.) of the oil droplets in these emulsions, as determined by microscopy, was 13 ± 4 µm (*n* = 402) for MOE and 15 ± 5 µm (*n* = 436) for SOE (Supplementary Fig. 1b).

To ensure accurate drug administration, we verified that the emulsions can be dispensed precisely and consistently using a micropipette. This was confirmed by the low coefficients of variation observed when a fixed volume was repeatedly pipetted (Supplementary Fig. 1c).

Both MOE and SOE emulsions improved the voluntary consumption of oil, with more than 60% of the mice drinking the sweetened solutions within the first 60 s, while only 10% of the mice voluntarily consumed oil without any additives. After 3 days of training, the fraction of animals that voluntarily consumed the offered solutions increased to more than 75% for MOE and SOE, while it remained unchanged for oil (Fig. 1d). Training with the sweetened emulsions shortened the manipulation time for most mice to just 10–30 s per administration (Fig. 1e and Supplementary Fig. 2a). Given that it takes some time for the mice to become aware of the pipette tip, the actual drinking time is even shorter. Training is thus important since it results in shorter handling times, which is beneficial for the mice and minimizes overall experimental procedure times.

### Sweetened emulsions and training encourage TAM consumption

To test whether the sweetened emulsions also improved TAM consumption, mice were trained for 3 days with MOE or SOE and, on the fourth day, received MOE or SOE containing TAM (referred to as MOE-TAM and SOE-TAM, respectively) at a final dose of 80 mg/kg. Similarly, control mice were pipette-fed with oil (training) and then with oil containing TAM (OIL-TAM) at a final dose of 80 mg/kg. We observed that 75% and 60% of the mice voluntarily consumed MOE-TAM and SOE-TAM, respectively, versus 25% for OIL-TAM solutions (Fig. 1d). Interestingly, when the oil was sterilized by filtration instead of heat treatment, the mice more readily consumed the sweetened emulsions (Supplementary Fig. 2a), suggesting that heating the oil affected the taste of the emulsions. Indeed, with filtered oil, the fraction of mice that voluntarily consumed the emulsions during training and subsequent administration of TAM increased to 100% (Fig. 1e and Supplementary Fig. 2a–c).

The use of transparent tunnels in husbandry and experiments has been described as beneficial to animal welfare^34^. Therefore, we incorporated tunnel handling into our procedures. Although we found that the tunnels were helpful for removal from the home cage, weighing, identification and transfer to a cage grid for feeding (Supplementary Fig. 3), the animals could not be motivated to drink the emulsions from within a tunnel.

Taken together, the use of sweetened oil-in-water emulsions motivates mice to voluntarily consume TAM after at least 2 days of training.

### Efficacy of pipette-administered MOE-TAM is noninferior to that of gavaged or IP-injected OIL-TAM

Having shown that sweetened emulsions encourage voluntary consumption of TAM, we next compared the efficacy of TAM administered with a micropipette using SOE and MOE formulations with that obtained after OG or IP injection of OIL-TAM. The capacity to induce Cre-mediated recombination was analyzed using a mouse model where the CD4-CreER^T2^ knock-in strain^35,36^ is crossed with mice containing an inserted transgene, in which the VαJα exon of the alpha chain of an HY-specific T cell receptor (TCRα) is flipped into transcriptional orientation following Cre-mediated recombination. In the correct orientation, the complete TCRαβ is expressed on the cell surface of thymocytes owing to the presence of a conventional transgene in these mice coding for the corresponding HY-TCRβ chain^37^.

**Fig. 2:**
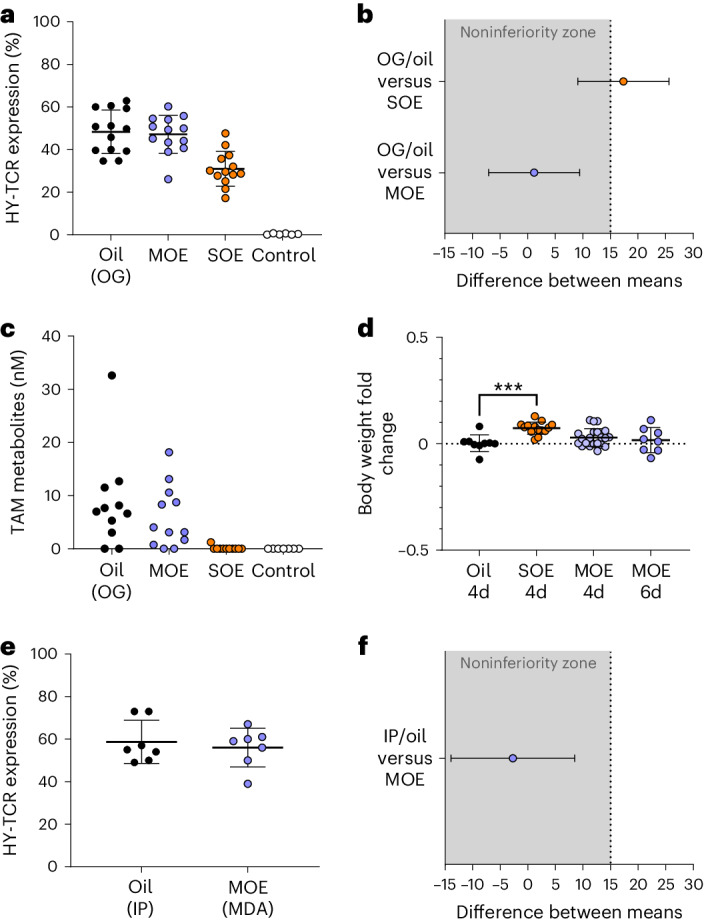
Efficacy of TAM-induced gene expression. Comparison of CreER^T2^-dependent HY-TCR reporter expression in thymocytes of adult female mice, 40 h after treatment with a single dose of 80 mg/kg TAM. Mice were either treated with TAM dissolved in oil via OG and IP injections or pipette-fed with oil-in-water emulsions made with sweetened milk (MOE) or syrup (SOE). **a**, The percentage of HY-TCR-expressing thymocytes was determined via flow cytometry as described in Methods. Shown are the means ± s.d. per treatment group (*n* = 13 per group). **b**, Noninferiority graph, depicting the 95% confidence interval (CI) of the difference between the mean of the OG-oil group and SOE group or MOE group of the results shown in **a**. The 95% CIs were computed using Dunnett’s confidence interval formula following the ANOVA. The noninferiority margin ‘*d*’ was set at 0.18 (1.5 times s.d. of the control OG-oil group). **c**, TAM metabolite concentrations in the serum of mice described in **a** were assessed in vitro using MEFs from R26-CreER^T2^-Ai14 mice. **d**, The fold change in body weights relative to the oil group following daily administration of the indicated formulations for 4 or 6 days. Group sizes: OG-oil, *n* = 9; SOE, *n* = 14; MOE, *n* = 21. Depicted are means ± s.d. ***P = 0.0006 according to one-way ANOVA followed by post-hoc Dunnett’s multiple comparison test. **e**, The percentage of HY-TCR-expressing thymocytes induced after IP injection of OIL-TAM or pipette feeding with MOE-TAM (*n* = 7). MDA, micropipette-guided drug administration. **f**, Noninferiority graph, depicting the 95% CI of the difference between the means of the IP oil group and MOE group of the results shown in **e**. The controls shown in **a** and **c** were HY-TCR knock-in mice trained (3 days) with MOE but not treated with TAM (*n* = 6). Source data

Using these so-called HY-switch mice (mice that will express the HY-specific TCR on thymocytes when treated with TAM), we first performed a noninferiority analysis to determine if the new formulations are not worse than—or ‘noninferior to’—the standard OG or IP treatments^38^. The experiment was prospectively powered (Methods) on the basis of preliminary data from HY-TCR expression induced after conventional TAM treatment (OG of OIL-TAM). Induction of HY-TCR expression (% HY-TCR-positive cells) on thymocytes was analyzed 40 h after pipette feeding the mice with a single dose of MOE-TAM, SOE-TAM or, as a control, OIL-TAM administered via OG (Fig. 2a–d) or IP injections (Fig. 2e,f). With each treatment, mice received 80 mg of TAM per kilogram of body weight. For the emulsions (MOE-TAM and SOE-TAM), such an accurate and mouse-tailored dose administration was achieved simply by changing the volume dispensed by the micropipette. However, similar accurate dosing is not possible with the syringes that are typically used for IP injections or that are connected to the gavage needle. Since accurate dosing was essential to compare the efficacy of the different methods correctly, we prepared weight-adjusted OIL-TAM solutions for each mouse in the OG and IP groups, of which the corresponding mouse then received 200 µl (OG) or 100 µl (IP).

The efficacy of the different TAM formulations in inducing HY-TCR gene expression in thymocytes was quantified by flow cytometry. We first compared feeding TAM in MOE or SOE formulations with the OG of OIL-TAM, for which the results of three independent experiments were combined to achieve the number of mice per group (*n* = 13) required for noninferiority testing (with each experiment comparing equivalent group sizes). The results showed that the efficacy of induction of HY-TCR expression (mean of the fraction of cells that express the HY-TCR) in the MOE-TAM group was similar to that of the OG-OIL-TAM group (47% versus 48%) (Fig. 2a), whereas in the SOE-TAM group this expression was lower (31%). A noninferiority analysis confirmed that pipette-fed MOE-TAM but not SOE-TAM is noninferior to standard OG-OIL-TAM (Fig. 2b). As a negative control, mice were trained for 3 days with MOE but not treated with TAM (*n* = 6). In these mice, as well as in untreated mice (data not shown), HY-TCR expression in the thymus was undetectable (Fig. 2a). Interestingly, SOE- but not MOE-treated groups significantly gained weight compared to the control group receiving oil only (Fig. 2d, *P* < 0.0006 based on a one-way analysis of variance (ANOVA) followed by a post-hoc Dunnett’s multiple comparison test). This weight gain might result from the higher sugar content of SOE (54%) as compared to MOE (9%) (Supplementary Table 1). Based on the lower TAM efficacy and, additionally, the increase in body weight in the SOE group (Fig. 2a–d), we used only sweetened milk emulsions as a vehicle for our subsequent experiments.

Since TAM is also frequently administered via IP injections^39^, we similarly compared the efficacy of HY-TCR induction after feeding MOE-TAM with that induced by standard IP injection of OIL-TAM. As seen for OG, we obtained comparable induction of HY-TCR expression after MOE-TAM feeding and IP injections, with similar means of HY-TCR-expressing cells in both groups (56% versus 59%) (Fig. 2e). Accordingly, the noninferiority analysis (Fig. 2f) showed pipette feeding of MOE-TAM to also be noninferior to IP injection of OIL-TAM.

Finally, we observed that young, prepuberty (5 weeks) mice can successfully be trained and pipette-fed with MOE-TAM (Supplementary Fig. 4), offering a welcome refined administration method for smaller animals instead of IP injections or OG. When compared to adult mice, the young mice were shyer and overall needed a slightly longer time to consume the offered volumes during training and MOE-TAM consumption (Supplementary Fig. 4a). Despite this observation, the success rate in feeding and TAM efficacy in such young animals was similar to that of adult mice (Supplementary Fig. 4b). Finally, we did not observe any differences in feeding behavior (Supplementary Fig. 4a) or efficacy of TAM gene induction (not shown) between male and female mice.

Changes in levels of blood corticosterone (CORT) are often used to measure stress responses in rodents^40^. Prior studies have suggested that repeated pipette feeding induces a lower CORT response than traditional gavage or injection routes^7,8^. To test this stress response in the different application methods, we compared blood CORT levels 30 min after the mice were subjected to IP injection, voluntary and restrained pipette feeding, or OG (Supplementary Fig. 5). To exclude a direct effect of TAM on CORT levels due to its anti-estrogenic activity^41^, the mice were only treated with the respective vehicles. Compared to the control group, all treatments resulted in higher CORT levels, with the highest increase observed for the OG group, making OG the least animal-friendly method with respect to stress response. Among the other treatments, restrained MOE feeding and IP injections showed similar effects on plasma CORT levels, suggesting that these treatments are equally stressful. Changes in blood glucose were also reported to be a stress marker^42,43^. However, we did not detect substantial differences in glucose levels 30 min after vehicle application between any of our studied application routes or control mice (data not shown).

Taken together, our results show that only MOE-TAM is equally efficient in inducing CreER^T2^ activation when compared to OG and IP injection and offers an animal-friendly alternative to both OG and IP injections for TAM administration. The method can also be used for the treatment of young and, thus, smaller animals. An additional advantage of the micropipette administration method is that it allows for accurate dosing of TAM without the need to make separate OIL-TAM solutions for each mouse, thereby also improving experimental conditions and reproducibility.

### Serum concentrations of TAM metabolites correlate with CreER^T2^-induced gene expression

CreER^T2^ activation in mice after TAM treatment is primarily mediated by its major bioactive metabolite 4-OH TAM^44,45^, since TAM itself is a poor inducer of CreER^T2^ (refs. ^44,46,47^). Thus, Cre-mediated recombination is highly dependent on the efficient first-pass metabolism of TAM in the liver^22,44,45,48^. To directly assess serum concentrations of bioactive TAM metabolites in the different treated mice as a measure of first-pass TAM metabolism and, therefore, compare the pharmacological properties of the different formulations, we developed an in vitro assay based on mesenchymal embryonic fibroblasts (MEFs) isolated from R26-CreER^T2^-Ai14 mice. When these MEFs are exposed to 4-OH TAM, a constitutively expressed recombinant CreER^T2^ protein mediates the removal of a *loxP*-flanked STOP cassette, resulting in the expression of a red fluorescent tdTomato protein. As shown by time-lapse microscopy and flow cytometry (Supplementary Fig. 6a–c), induction of tdTomato expression in these cells is time and dose dependent.

To test if MEFs can be used to quantify serum TAM metabolites, we compared serum metabolite concentrations obtained with R26-CreER^T2^-Ai14 MEF to those obtained with an EU-approved assay^49^, known as the LUMI-CELL ER assay^50^. This latter cell culture-based assay was developed for the quantification of ER agonists^50,51^, but it can also detect ER antagonists such as 4-OH TAM^51^. We used sera collected from C57BL/6 mice, 6 h after OG treatment with OIL-TAM (40 or 80 mg/kg) and compared the results obtained with both assays. Although the principle of TAM metabolite detection is different, both assays gave comparable concentrations for each serum (Supplementary Fig. 6d), confirming that the R26-CreER^T2^-Ai14 MEF system can indeed be used to determine and compare serum TAM metabolite concentrations. Since reporter expression in the MEF directly measures CreER^T2^ activation, we used this assay to compare bioactive serum TAM metabolite concentrations from CreER^T2^ mice that were treated with the different TAM formulations.

In line with the induction of HY-TCR expression (Fig. 2a,b), treatment with OIL-TAM by OG or pipette feeding with MOE-TAM resulted in comparable serum concentrations of TAM metabolites (Fig. 2c), confirming that first-pass metabolism of TAM administered in sweetened milk emulsions is comparable to that obtained with conventional OG. Serum TAM metabolite concentrations typically peak at 6–7 h after TAM administration and then rapidly decrease over the next 24–48 h (ref. ^52^). Accordingly, at the time point when the mice were killed, which was optimal to evaluate gene expression, only low metabolite concentrations (nanomolar range) were observed (Fig. 2c). None of the mice fed with SOE-TAM had detectable serum TAM metabolites at the time of analysis, in line with the lower levels of TCR induction observed with this formulation.

Together, our results indicate that voluntary consumption of MOE-TAM formulations is as efficient as conventional OG or IP treatments regarding reporter gene induction and generation of serum TAM metabolites (compared to OG only). By contrast, TAM in syrup emulsions (SOE-TAM) is less effective compared to both OIL-TAM (administered via OG) and MOE-TAM formulations and, in addition, results in significant increases in body weight.

Thus, while both sweetened milk and syrup-based emulsions encourage mice to consume TAM offered with a micropipette voluntarily, only MOE offers an acceptable alternative method to OG or IP injections.

### Repeated administration of MOE-TAM increases the induction of reporter gene expression

**Fig. 3:**
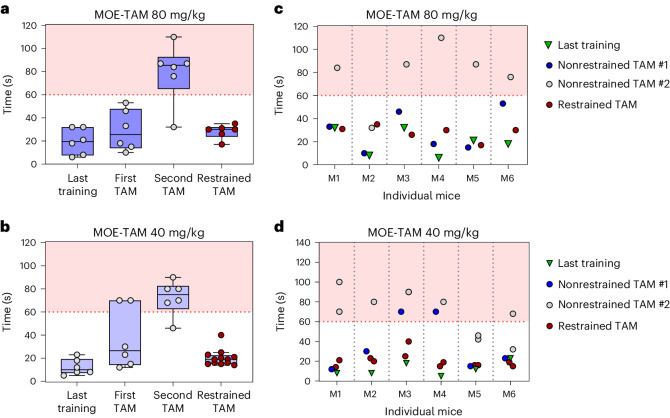
Repeated administration of TAM using MOE. **a**–**d**, Adult male and female HY-switch mice were trained for 3 days before MOE-TAM treatment. Consumption time for the last training, first (#1) and second (#2) TAM treatment was recorded and displayed as described in Fig. 1e. For subsequent MOE-TAM treatments, mice were immediately restrained (red dot plots). Treatment with 80 mg/kg (**a** and **c**) and 40 mg/kg (**b** and **d**) TAM in MOE. **c** and **d** show the consumption time for individual mice (M1 to M6) for data shown in **a** and **b**, respectively. Mice were either offered the formulations without being restrained for the first 60 s (gray or blue dot plots) or immediately restrained (red dot plots). For **a** and **b** minimum and maximum values, interquartile range and median are depicted as Tukey box plots with individual data points shown as scatter dot plots. Source data

In the experiments described above, HY-switch mice were treated with only one dose of TAM to induce a cohort of TCR-expressing cells. While such one-time treatment is also used for other mouse models^53^, most in vivo studies involving CreER^T^ or CreER^T2^-mediated gene expression require repeated treatments to ensure sufficient genetic recombination in target tissues or cells^11,35^. Therefore, we tested the efficacy of treating animals with MOE-TAM once every 24 h for five consecutive days, a treatment regimen recommended for many CreER^T2^-based mouse models^11,39^. As observed before, the mice voluntarily consumed MOE-TAM on the first day. However, they were reluctant to readily consume a second dose the next day and instead required restraint after 60 s (as shown in Fig. 1c and Supplementary Video 2) to finish drinking the offered volume (Fig. 3a,c).

Since split dosage treatment of mice, twice a day, was shown by others to result in similar recombination frequencies as a full TAM dose^54^, we tested whether this apparent aversion could be overcome by halving the TAM dose to 40 mg/kg. Yet also here, most mice refused to voluntarily consume a second serving of this lower amount, although they had readily drunk the first dose the day before (Fig. 3b,d). Hence, feeding the mice twice with half the intended dosage does not overcome the observed avoidance and is thus not a solution. Interestingly, we noticed that, for both high and low MOE-TAM dosage formulations, once the mice were restrained (that is, after 60 s), they quickly drank the formulation (Fig. 3a,b), despite having shown little or no interest in it while sitting on the grid. Therefore, we tested whether mice would also rapidly consume additional treatments if they were immediately held by the scruff. Indeed, mice consumed additional treatments without hesitation while being held by the scruff (Fig. 3a–d, red dots), independently of the dose offered (40 mg/kg or 80 mg/kg). Therefore, feeding daily doses of MOE-TAM for at least 5 days is possible but is best performed by gently restraining the mice from the second dose onwards.

**Fig. 4:**
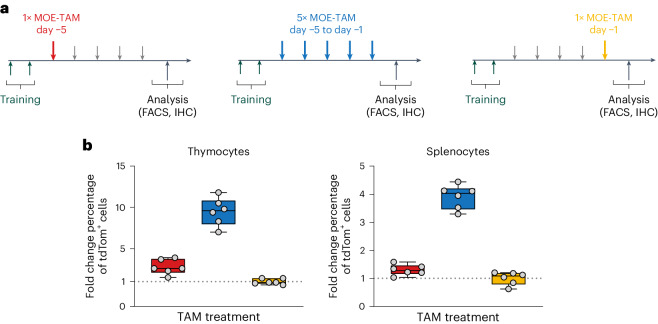
Multiple administration of TAM in MOE. **a**, Induction of tdTomato expression was assessed in adult R26-CreER^T2^-Ai14-tdTomato mice after training (green arrows) and TAM treatments as indicated. Mice were either offered MOE-TAM (20 mg/kg) once a day for 5 days (blue) or only once on day −5 (red) or day −1 (yellow) (*n* = 6 per group). Mice that did not receive TAM on the indicated days were offered MOE vehicle only (short gray arrows). On day 0, the mice were euthanized and analyzed using flow cytometry (fluorescence-activated cell sorting, FACS) and immunohistochemistry (IHC). **b**, The percentages of thymocytes and splenocytes expressing tdTomato were determined by flow cytometric analysis. Tukey box plots depict fold change compared to the fraction of positive cells detected in mice treated only once on day −1 and include minimum and maximum values, interquartile range and median with individual data points overlayed as scatter dot plots. Source data

Given that mice readily consume sweetened MOE-TAM emulsions for at least 5 days, we evaluated the efficacy of such repeated MOE-TAM treatments using R26-CreER^T2^-Ai14 reporter mice, the same mouse line that was used to derive the R26-CreER^T2^-Ai14 MEFs for the in vitro assay. This mouse strain is more suitable for monitoring long-term TAM treatment owing to the ubiquitous and additive expression of the tdTomato reporter. We compared three groups of mice (Fig. 4a), namely animals treated daily with MOE-TAM for 5 days (day −5 to day −1) and two other groups that received only once MOE-TAM, on either day −1 (last day) or day −5 (first day). The expression of tdTomato was evaluated by flow cytometry in thymocytes and splenocytes (Fig. 4b) and by fluorescence microscopy of spleen sections (Supplementary Fig. 7). These results show increased induction of tdTomato reporter expression in both thymocytes and splenocytes after repeated feeding of MOE-TAM compared to one-time treated mice, reflecting efficient repeated delivery of TAM and increased induction of CreER^T2^ activation with this method.

In conclusion, our results show that repeated administration of MOE-TAM results in greater reporter induction compared to a single MOE-TAM treatment but requires holding the mice by the scruff, except during training and the first TAM treatment.

## Discussion

Animal studies are important for the advancement of our understanding of animal and human physiology, as well as for the development of new therapies^55–57^. However, some current laboratory practices, even when used for years as standard procedures, are suboptimal regarding animal welfare. Physiological changes associated with discomfort or stress have been reported to interfere with and confound experimental outcomes^24–27^, potentially compromising the reliability and reproducibility of experimental results. Over the past few years, new procedures have been explored to reduce stress and injuries related to drug administration. Such methods include voluntary drug feeding of rodents using palatable vehicles (for example, sucrose water, peanut butter, tablets, jam or jelly)^58–61^. Unfortunately, these methods do not allow accurate drug delivery, potentially resulting in over- or underdosing^10,62^, or require individual housing, which was shown to inflict stress on animals^63^.

A promising drug administration method that allows accurate drug delivery with minimal or no induced stress responses is the micropipette-guided drug administration^7,8^. Although this method was shown to replace IP and OG with similar efficacy for the drugs tested, up until now, it has only been reported for the administration of water-soluble drugs. In the current study, we show that mice can be encouraged to voluntarily consume water-insoluble drugs such as TAM using a micropipette by administering the drug in sweetened ‘oil-in-water’ emulsions. The mice eagerly consumed both sweetened milk- and syrup-based emulsions, but only feeding TAM in sweetened milk emulsions resulted in CreER^T2^-mediated reporter expression that is statistically noninferior to OG or IP injections of OIL-TAM solutions. Stable oil-in-milk emulsions were obtained by a simple high-energy homogenization method using two connected syringes^33^, which is cost-effective and readily available to most research groups. Although we did not extensively explore alternative methods, more expensive professional homogenizers could allow more automated processing^64^. Larger volumes of MOE can be prepared for training and be frozen and thawed without coalescing (not shown). However, this may not be possible for emulsions containing TAM, since TAM tends to precipitate at low temperatures. Occasionally, CreER^T^-based mouse models are treated with 4-OH TAM instead of TAM^53^, which is also water insoluble and could thus also be administered using MOE formulations, although we have not tested this possibility.

Based on our observations, we recommend training mice for at least 2 days to obtain efficient consumption of MOE-TAM solutions, possibly because rodents are neophobic toward new foods^65,66^ and need to be first habituated to drinking from the pipette tip and/or to the taste of the formulations. Our attempts to directly feed the mice with MOE-TAM without prior training mostly failed, even when holding the mice by the scruff, and resulted in a loss of formulation. Sterilizing the oil by filtration instead of heat treatment further improved consumption of the emulsions, suggesting that changes in taste can affect palatability. The method is also affected by the preferences of individual mice (Fig. 3c,d), as reflected by differences in consumption times. Long-term daily treatments using pipette feeding of sweetened formulations have been reported for water-soluble drugs^7,8^. We show here that repeated daily pipette feeding of MOE-TAM is also possible, albeit only when the animals are gently held by the scruff after the first dose of TAM, suggesting the mice acquired a mild taste aversion or avoidance^58,67^. Our observations are in line with studies in rats, which showed that TAM treatment results in a shift in taste palatability and avoidance behavior rather than TAM aversion^68^. Also, patients treated with TAM have reported taste changes^69^, including metallic or bitter taste in the mouth or changes in the flavor of certain foods. Although we did not explore treatments longer than 5 days, we expect that additional daily MOE-TAM administrations will also be possible.

Changes in blood CORT levels suggest that restraining mice for pipette feeding is as stressful for the mice as holding them to inject oil in the peritoneum; however, both methods are less stressful than OG and, thus, better alternatives. Still, pipette feeding is more animal-friendly than IP injections; not only are injections prone to accidental injuries, but also oil injected in the peritoneum was shown to cause localized inflammation in mice^12^. In addition, in many legislations, pipette feeding may be considered as an animal experiment without harm to the animal (for example, severity 0 in Switzerland), which is an important factor for the outcome of the harm–benefit analysis. For young and/or small animals, especially, for which IP injections and OG are both more challenging and prone to resulting in complications, administering drugs like TAM in palatable formulations provides an appealing alternative to IP injections or OG.

Whereas mice readily accepted drinking both SOE-TAM and MOE-TAM formulations, the reduced serum concentrations of TAM metabolites and gene induction in mice treated with SOE-TAM suggest that substances in syrup potentially affected TAM efficacy. By contrast, treating mice with MOE-TAM resulted in CreER^T2^-mediated gene expression that is equivalent to conventional OG or IP injections of OIL-TAM, indicating that the TAM administration and first-pass metabolism are unaffected by sweetened milk. Administering MOE-TAM formulations for at least 5 days, which we confirm to result in enhanced reporter gene recombination and expression when compared to treating the mice only once, will allow successful application of this method to a larger number of different CreER^T2^ mouse models.

The administration of drugs, including TAM dissolved in oil, by pipetting directly into the mouths of mice has been previously reported^70,71^. However, these methods did not rely on palatable drug formulations or voluntary consumption by mice. Instead, the mice were forced to swallow the solution by pressing the pipette tip against the hard palate^70^ or behind the diastema of the mouth^71^. While this method is likely to be more stressful than our voluntary administration method and might risk damaging the mouse, efficient reporter gene induction was reported when TAM was administered in this manner^71^. Nevertheless, TAM delivery seems to be suboptimal with this method because consistent results were obtained only with very high TAM doses (7.5–10 mg per mouse, corresponding to 300–400 mg/kg)^71^. Differences in CreER^T2^ strains used in that study compared to strains used in our experiments could explain the differences in TAM doses required. However, in this previous study, no direct comparison was made with conventional OG or IP administrations^71^. It was also neither shown nor discussed if mice could be treated with more than one dose with this method. By contrast, our study demonstrates that the efficacy of palatable MOE formulations is statistically noninferior to that obtained after OG or IP injections of identical TAM doses. Furthermore, we obtained consistent induction of reporter expression within groups of mice, even with MOE-TAM doses of as low as 0.5 mg TAM per mouse (20 mg/kg) (Fig. 4). In our experience, any formulation that is not actively licked up by the mice will result in loss of material, leading to variable doses and, thus, variable experimental outcomes. We observed that feeding only oil is very inefficient. Drops of oil tend to rapidly spread in the fur around the mouths of the mice, and most mice just kept the oil in their mouths without drinking it, suggesting that the drinking of solutions cannot be forced, even when the mice are held by the scruff.

Although treatment with palatable formulations presents many advantages, there are also some limitations to consider. Of note, the respective drug could interact with the vehicle, thereby affecting treatment efficacy, as we observed for syrup and TAM. Depending on the drug tested and the experimental readouts, the sweetening substances in the formulations could compound experimental outcomes. For example, MOE formulations would be incompatible with CreER^T^ models that aim to study key genes involved in glucose metabolism or insulin sensitivity and resistance^72,73^. Furthermore, the vehicle itself may contain activities relevant to the measured variable. On the other hand, feeding drugs in MOE formulations might be more suitable for metabolic studies as compared to conventional OG or IP injections of oil, since MOE solutions have a lower total energy content than oil and less sugar than syrup-based emulsions (Supplementary Table 1).

Finally, pipette feeding may not work when a drug cannot be made palatable by sweetening, for example, if it has a strong, unpleasant taste. For such drugs or those that induce strong adverse effects, or for CreER^T^ models where modulation of the target gene would induce aversion, OG might still be necessary. We observed that mice showed a mild aversion/avoidance after the first treatment with MOE-TAM, requiring holding the mice by the scruff for feeding additional daily dosages. Such gentle restraint could interfere, for example, with behavioral studies or induce stress levels that could affect experimental results. However, because the mice still voluntarily consumed MOE-TAM, pipette feeding is still preferable to conventional OG or IP injections, which also require restraint and, in addition, are prone to accidental injuries. While our method requires additional time and effort for training the mice, we typically combine the second training session with the weighing of the mice to determine the appropriate amount of the drug to administer to achieve the required dose.

Alternative methods to prepare TAM-containing microemulsions have been reported as replacements for tablets to treat patients with breast cancer. However, these microemulsions did not contain sweet substances, were made with detergent-based emulsifiers and were either tested solely in vitro^74^ or administered to tumor-bearing mice via OG^75^.

There is little consensus in the scientific community on the TAM dosage necessary to induce sufficient CreER^T2^-activation in mice^12,54^. The reasons may lie in the variable exposure of different target tissues, differences in locus accessibility for recombination, kinetics in gene expression and, finally, protein stability. In most published experiments, typically all mice receive the same volume of TAM through OG or IP injections regardless of body weight, prompting the preferential use of high dosages to ensure complete recombination. While administered volumes can be adjusted to some extent with the syringes used for OG or injections, micropipette administration of MOE-TAM formulations allows for more accurate treatments over a wide range of dosages by adjusting both the administered volume as well as the concentration of TAM in the sweetened milk emulsions. In our experience, stock solutions of 80 mg/ml of TAM in oil, that is, a final 40 mg/ml of TAM in the MOE emulsion, is the recommended maximum concentration to use. Higher TAM concentrations could result in precipitation of TAM in the emulsions, especially below room temperature, which will not only affect treatment accuracy but could also cause adverse effects in mice^76^. High TAM concentrations such as 200 mg/kg are in any case not recommended as they do not increase the rate of recombination and can lead to metabolic stress and increased lethality^54^. Using the pipette-administration method, we successfully administered TAM doses from 20 mg/kg to 120 mg/kg with MOE-TAM volumes ranging from 30 µl to 120 µl using a 200 µl micropipette, including with mice as young as 5 weeks.

Administration of TAM by chow or drinking water has been used for some experimental applications but has the disadvantage that the timing and the amount of consumed drug are not controlled^10,62^, that the mice reduce their food or water intake^58,62^ or that single-housing of the animals is required^5,77–79^. Single housing of rodents is a known stressor with physiological consequences^27,80^ and is thus strongly discouraged.

Several studies have suggested that a more humane approach to reducing pain and distress experienced by laboratory animals will positively impact behavioral and physiological processes and, therefore, reduce variability in experimental data^24–27^. Thus, reducing pain and distress will not only benefit animal welfare but will also, because of reduced experimental variability, help conduct more reliable and robust experiments and reduce the number of experimental animals required per experiment.

Taken together, dosing with the micropipette method is a valuable, more animal-friendly alternative to more invasive methods such as OG and IP injections. Because administered volumes can be easily adjusted with the micropipette, dosing is also more accurate, and holding the mice by the tail or the scruff can be performed with little training of the experimenter.

We used TAM as an archetype drug to evaluate a noninvasive administration procedure for water-insoluble drugs based on voluntary consumption. This new method adds to a growing number of applications that aim to replace more invasive administration routes, such as OG or injections, with animal-friendly methods that are equally accurate in dosing, timing and outcome^7,8^.

## Methods

### Animals

All mouse strains used in this project were of C57BL/6J genetic background. HY-switch mice were obtained by crossing the following strains: CD4-CreER^T2^(B6.CD4^tm1(Cre/ERT2)ThBu^)^35^, HYβtg mice (B6.Tg(Tcrb)93Vbo)^37^ and HYα^sn^ (B6.TCRA^tm2ThBu^ (unpublished)). The resulting HY-switch mice express the CreER^T2^ protein under the control of the CD4 promoter and have a *loxP*-flanked HY-TCR VαJα exon inserted into the TCRα locus so that expression of HY-TCR is only possible after Cre-mediated inversion and the presence of the HY-TCRβ transgene (HYβtg). The TCRδ locus was deleted by Cre-mediated recombination in the germline. Only female HY-switch mice (10–18 weeks of age) were used for the noninferiority study since HY-TCR-expressing thymocytes are specific for the male HY self-antigen and are readily eliminated in male mice due to negative selection^81,82^. The tdTomato reporter mice used in this study were generated by crossing R26-CreER^T2^ mice (B6.129-Gt(ROSA)26Sor^tm1(cre/ERT2)Tyj^/J, JAX stock #008463)^83^ and Ai14 mice (B6.Cg-Gt(ROSA)26Sor^tm14(CAG-tdTomato)Hze^/J, JAX stock #007914)^84^. Both males and females between 10–18 weeks of age were used. All mice were bred in-house under specific and opportunistic pathogen-free conditions and used for experiments in a conventional sterile animal facility at the Laboratory Animal Services Center of the University of Zurich in the absence of Federation of European Laboratory Animal Science Associations-listed pathogens and group-housed in individually ventilated NexGen type II long cages (Allentown) with a maximum of five animals per cage. Cages were autoclaved with wood chips (Lignocel Select, J. Rettenmaier & Söhne) as bedding material and a mouse house (red transparent, polycarbonate, Zoonlab GmbH). Paper tissues (Uehlinger AG) and paper fibers (Arbocel Crinklets Natural, J. Rettenmaier & Söhne) were used as enrichment. The breeding food (3802 extrudate for specific and opportunistic pathogen-free and 3336 extrudate for sterile rooms, KLIBA NAFAG) was supplied ad libitum. The animals were kept under a controlled light cycle (14/10 h), room temperature (21 °C) and room humidity (45–60%). Health monitoring using sentinels on dirty cages was performed quarterly in accordance with Federation of European Laboratory Animal Science Associations guidelines. Personnel wore masks, bonnets, gloves, dedicated clothes, disposable overalls and dedicated shoes.

Mice were acclimated for at least 7 days before experimental use. All experimental procedures described were approved by the Cantonal Veterinarian’s Office of Zürich, Switzerland, and the district government of Cologne, Germany (respective license numbers ZH107/2020, ZH063/18, 50.203.2-K 13).

### In vitro TAM assays

TAM metabolite detection was performed with the experimenter blinded to the serum samples. The LUMI-CELL ER assay is based on the VM7Luc4E2 cell line, generated by Prof. M. S. Denison (University of California, Davis) by transfection of human MCF7 breast cancer cells with the pGudLuc7 plasmid^50,85^. The luciferase (Luc) reporter in VM7Luc4E2 cells responds in a dose-dependent manner to ER agonists or antagonists (for example, 4-OH TAM)^49,50,85^. VM7Luc4E2 cells were cultured in estrogen-free medium as published^50,86^. Estrogen-depleted VM7Luc4E2 cells were seeded at 6.6 × 10^4^ cells per well in 100 µl assay medium in 96-well plates and cultured at 37 °C with 5% CO_2_. Twenty-two hours later, per well, 100 µl of medium was added containing estrogen (0.1 nM), 4-OH TAM or diluted mouse sera (final concentration 15%). Each sample was tested in triplicate. After 22 h, cells were washed twice with Dulbecco’s phosphate-buffered saline, and luciferase activity was recorded using the Promega Luciferase assay system (Promega #E4030) according to the manufacturer’s instructions and as published^50^ using a TECAN reader (SPARK TECAN). The standard curve of inhibition of estrogen-induced Luc expression in response to serial dilutions of 4-OH TAM (5–80 nM) was fitted by a sigmoidal curve (*R*^2^ > 0.9) according to the Prism 4-parameter fit algorithm (4PL) and used to calculate the concentration of TAM metabolites for each tested mouse serum (Prism v9.2.0, GraphPad).

For the CreER^T2^-Ai14 reporter assay, MEFs were isolated from individual R26-CreER^T2^-Ai14 mice and cultured as previously described^87^. Selected clones were immortalized by transfection with the plasmid pBSSVD2005, coding for the large T oncogene SV40 (SV40 1: pBSSVD2005 was a gift from David Ron; Addgene plasmid #21826; http://n2t.net/addgene:21826; RRID:Addgene_21826), using LipofectamineTM LTX (Invitrogen #L3000-008) according to the manufacturer’s instructions and selected using a protocol from Heather P. Harding^88^. To detect serum TAM metabolites, MEFs were seeded in 24-well plates (Falcon #353047) at 5 × 10^4^ cells per well in 300 µl growing medium and cultured at 37 °C with 5% CO_2_. After 4–5 h, 100 µl of diluted mouse serum (final 15%) or serial dilutions of 4-OH TAM (1–30 nM 4-OH TAM) were added to duplicate wells. At the time points indicated in the figures, cells were washed and detached with trypsin and tdTomato expression was measured with a BD LSR Fortessa II (BD). Results were analyzed using the FlowJo v10.4 Software (BD Life Sciences). The percentage of tdTomato-positive cells in each well was determined by manual gating, as indicated in Supplementary Fig. 6b. The standard curve of responses (average of duplicate wells) to serial dilutions of 4-OH TAM was fitted by a sigmoidal curve (*R*^2^ > 0.9) according to the Prism 4-parameter fit algorithm (4PL) and used to calculate the relative concentration of metabolites of TAM for each mouse serum (Prism v9.2.0 GraphPad).

For time-lapse microscopy, MEFs were seeded in eight-well imaging chambers (Ibidi #80826) and treated with 7 nM 4-OH TAM or 70 nM 4-OH TAM, or left untreated. Selected fields (eight for each well) were imaged using an inverted widefield microscope (Zeiss, Axio Observer Z1) provided with temperature and CO_2_ control (37 °C and 5% CO_2_), every 20 min for a 23 h period with a 63× objective. Images were processed and analyzed using ImageJ software (v1.53c National Institutes of Health).

### Preparation of emulsions

Peanut oil (Sigma #P2144) was sterilized at 160 °C for 3 h or by filtering (0.22 µm Steriflip filter, Sigma #C3238). TAM (Sigma #T5648) was dissolved in 100% ethanol (Avantor #32221) at 100 mg/ml and mixed with an identical volume of peanut oil^35^. The oil/ethanol/TAM solution was heated in a sonicator bath (Transsonic Digital D-7700 Elma) to 58 °C. A vacuum was applied to accelerate the evaporation of the ethanol. For each experiment, a fresh 100 mg/ml TAM in oil stock solution was prepared and used to make the different formulations as indicated.

MOEs for this study were prepared with sweetened condensed milk (Nestlé Milchmädchen—sweetened condensed milk with 54.7% sugar, 8.5% fat and 20.5% nonfat milk dry mass) diluted 1:2 with sterile double-distilled water (Ecotainer, Braun, #82479E-E). Other brands of condensed milk (for example, MIGROS Kondensmilch, Migros) are also suitable^7,8^. MOE emulsions were made using one volume of peanut oil and one volume of diluted milk, homogenized using the two-syringe method (also known as syringe extrusion) as published^33,89^ and detailed below. MOE-TAM emulsions were prepared with oil containing 50 mg/ml TAM (for treatments with 80 mg/kg) or 25 mg/ml TAM (for treatments with 40 mg/kg).

SOEs were prepared using two volumes of undiluted syrup (Coop, with 81% sugar, 40% berry juice from concentrate, acidifier E330, H_2_O and flavoring) and one volume of peanut oil. SOE-TAM emulsions were made as above, but with oil containing 75 mg/ml TAM (for treatments with 80 mg/kg).

All reagents and solutions were kept sterile. Both MOE and SOE emulsions were freshly made on the day of treatment by emulsification during 10 min at room temperature using 2 ml Luer-Lock syringes (Braun #4606701V) connected with a one-way Luer female-to-female adaptor (Cadence Science #6521IND).

Oil and emulsions were always dispensed using reverse pipetting^90^. The accuracy of pipetting emulsions was tested by repeated dispensing and weighing (microbalance) of a fixed volume (75 µl).

For determining the diameter of the oil droplets in the emulsions, the fluorescent dye Nile Red (Sigma #72485) was dissolved in peanut oil (final concentration 20 µg/ml). Fast Green (Sigma #F7258) to stain proteins (first dissolved in distilled water at 1 mg/ml) was added to the diluted milk or syrup (final concentration 60 µg/ml). SOE and MOE emulsions were prepared using solutions as described above and samples of each were diluted and placed on a glass slide, covered with a coverslip and imaged immediately using an inverted confocal microscope (Leica DMI6000 AFC, Model SP8)^91^. Fluorescence microscopy images of the emulsions were processed using ImageJ v1.53t^92^.

### Administering formulations to mice

To reduce handling stress^93^, transparent training tunnels (Zoonlab #3084094) were used as described^34^ for transferring and identifying the mice (Supplementary Fig. 3). Training and treatment of mice were as published^7^, with the following modifications. The formulations were offered to the mice using a variable volume type P200 PIPETMAN P (Gilson) with a sterile 200 µl graduated filtered plastic tip (TipOne #S1120-8810, Starlab). Mice were collected from the home cage, transferred to the grid of a second cage and gently held by the proximal part of the tail. The formulations were offered by dropwise expelling the liquid from the tip of the pipette as soon as the mouse started to drink. Consumption time was recorded from the moment the liquid was offered until all the liquid from the tip was consumed. If, after 60 s, the mouse had not drunk or had drunk only part of the formulation, it was restrained by gently grabbing the scruff of its neck and the remaining formulation was offered again. Total consumption time is the time before restraining plus, if applicable, the time to consume the remaining formulation with restraining. Treated mice were temporarily placed in a third cage and then returned to the home cage when all the mice of a cage were treated. For the treatment with TAM-containing emulsions, mice received a dispensed volume that was adjusted on the basis of the weight of the mice as recorded during the last training day. For OG, a weight-adjusted OIL-TAM solution was prepared for each mouse by diluting the same 100 mg/ml TAM stock solution used to make the MOE and SOE emulsions. The corresponding mouse then received 200 µl using a curved OG needle of 50 mm/18G (Finescience, #18061-50) attached to a 1 ml syringe. Nontreated control mice or mice treated with different formulations or dosages were kept in separate cages to prevent confounding effects due to coprophagy.

### Flow cytometer analysis of ex vivo cells

Mice were killed 40 h after the last TAM treatment, and single-cell suspensions were prepared from each thymus. For HY-switch mice, cells were stained with AQUA Zombie live/dead dye (BioLegend #423101) according to the manufacturer’s instruction and then surface stained for TCRβ (BioLegend #2629564), CD8α (BioLegend #2562610), CD4 (BioLegend #893330), TCR H-Y (eBioScience #466267), CD3ε (eBioScience #469315), NK1.1 (BioLegend #313312), CD19 (BioLegend #313641) and CD25 (BioLegend #313392) in the presence of an Fc receptor blocking antibody (BioLegend AB_1574973) during 30 min at 4 °C. For tdTomato expression, cells were stained with Zombie NIR live/dead dye (BioLegend #423105) and then surface stained for TCRβ, CD3ε, CD8 and CD25 as above, in addition to CD4 (BioLegend #100428). After washing, samples were acquired with an LSR II Fortessa cytometer (BD Biosciences) or an Aurora (Cytek) spectral flow analyzer and analyzed by manual gating using FlowJo v10.4 software (BD Life Sciences). For HY-switch cells, gates were set to exclude dead cells, doublets and lineage (NK1.1, CD19 and CD25)-positive cells. HY-TCR expression was analyzed by setting a gate based on cells from nontreated mice. For R26-CreER^T2^-Ai14 mice, a gate was set to exclude dead cells and doublets. tdTomato-expressing cells were quantified using a gate based on nontreated cells.

### Serum and plasma collection

Mice were killed, and blood samples were collected via cardiac puncture using 25G needles (Terumo #AN*2516R1). Blood was transferred to serum separator tubes (BD Microtrainer #365968) for serum collection or heparin tubes (BD Microtainer #143455) for plasma and processed according to the manufacturer’s instructions. Individual sera and plasma were stored frozen at −20 °C and −80 °C, respectively, until analysis. Plasma CORT was measured by liquid chromatography–tandem mass spectrometry using Chromsystems KIT MassChrom Steroids (Chromsystems Instruments & Chemicals GmbH) on a QTRAP 6500 liquid chromatography–tandem mass spectrometry system.

### Tissue collection and microscopic analysis

Spleens were fixed in 4% formaldehyde (methanol-free paraformaldehyde, Thermo Scientific #28908) overnight at 4 °C, washed and transferred into phosphate-buffered saline containing 0.02% sodium azide and 20% sucrose and then cut with a microtome (CryoStar NX 50, Thermo Scientific) to obtain 8 µm tissue sections^94^ that were then stained for 10 min with 0.3 µM 4′,6-diamidino-2-phenylindole in Dulbecco’s phosphate-buffered saline (Thermo Scientific #62247). The sections were mounted with Fluoromount-G mounting medium (Invitrogen #00-4958-02) and imaged using an inverted confocal microscope (Leica DMI6000 AFC, Model SP8)^91^. Fluorescence microscopy images were processed using ImageJ v1.53t^92^ using identical settings for each channel.

### Statistics and data analysis

The noninferiority test was performed on the basis of published guidelines^38^. The 95% CI of the difference between means was derived using Dunnett’s test to correct for the multiple comparisons of multiple datasets to a single control dataset^95,96^.

As noninferiority margin, we chose 1.5 times the s.d. of the control group. One-way ANOVA followed by Dunnett’s test was applied when comparing multiple datasets to a single control dataset. All statistical analyses were computed using Prism (v9.2.0 GraphPad), and, where applicable, a *P* value <0.05 was considered statistically significant.

Tukey box plots in graphs show the minimum and maximum values (ends of the whiskers), interquartile range (length of the box) and median (line through the box) of sets of data. Individual data points are shown as circles.

Power calculations to determine the group size for the noninferiority test were performed in the statistical environment R, using a one-tailed *t*-test with a power of 0.8 and a significance level (*α*) of 0.025 (OG-MOE-SOE) or 0.05 (IP-MOE).

### Preregistration

Registered study in AnimalStudyRegistry.org: ‘THIVF—refinement of tamoxifen administration in mice.’ (10.17590/asr.0000306).

### Reporting summary

Further information on research design is available in the Nature Portfolio Reporting Summary linked to this article.

## Online content

Any methods, additional references, Nature Portfolio reporting summaries, source data, extended data, supplementary information, acknowledgements, peer review information; details of author contributions and competing interests; and statements of data and code availability are available at 10.1038/s41684-024-01409-z.

### Supplementary information

Supplementary Information Supplementary Figs. 1–7 and Table 1.

Reporting Summary

Supplementary Video 1 A representative example of voluntary consumption. Mice are collected from the home cage and placed on the new cage using a tunnel as shown in Supplementary Fig. 3. The mouse is gently held by the proximal tail with one hand and offered the solution using a 200 µl pipette with the other hand. Once the mouse shows interest in the tip/offered solution, small volumes are expelled according to how fast the mouse drinks the solution. After the mice consume the offered solution, they are either temporarily placed in a separate cage (when multiple mice need treatment) or returned directly to the home cage.

Supplementary Video 2 A representative example of restrained consumption. The mouse is gently restrained by holding the scruff of the neck with one hand while it is offered the formulation with the other. Similar as for voluntary consumption, small volumes are expelled from the pipette tip according to how fast the mouse drinks the solution. After the mice consume the offered solution, they are either temporarily placed in a separate cage (when multiple mice need treatment) or returned directly to the home cage.

### Source data

Source Data Fig. 1 Source data of graphs.

Source Data Fig. 2 Statistical source data.

Source Data Fig. 3 Source data of graphs.

Source Data Fig. 4 Source data of graphs.

## Data Availability

The data underlying examples and figures are available from the corresponding author or via Zenodo at 10.5281/zenodo.11978858 (ref. ^97^). Source data are provided with this paper.
