# Supplementary material for: Refined tamoxifen administration in mice by encouraging voluntary consumption of palatable formulations
Source: Lab Anim (NY). 2024 Jul 30;53(8):205–14. doi: 10.1038/s41684-024-01409-z (PMC11291282; doi:10.1038/s41684-024-01409-z)
Supplement: Supplementary file 1 — Supplementary Figs. 1–7 and Table 1. [file 41684_2024_1409_MOESM1_ESM.pdf]

lab animal

Supplementary information

https://doi.org/10.1038/s41684-024-01409-z

Refined tamoxifen administration in mice by encouraging voluntary consumption of palatable formulations

In the format provided by the authors and unedited

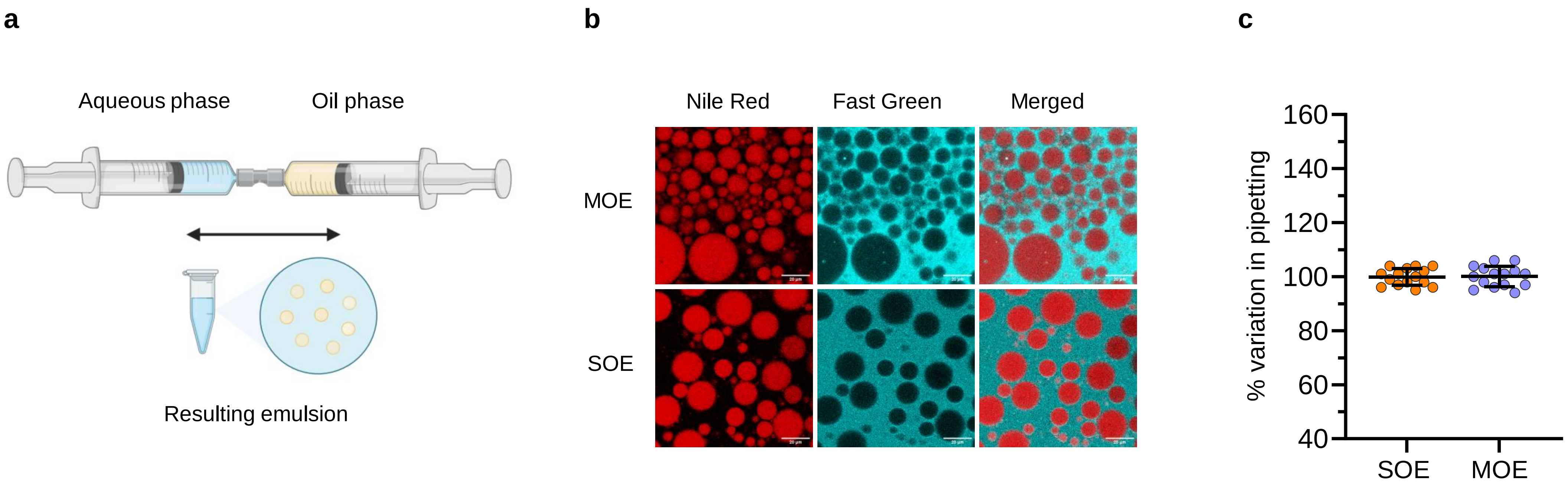

Figure S1: Oil-based emulsions as vehicles for TAM administration. a, The 2-syringe homogenization method involves connecting two syringes via a suitable adapter. The two solutions (aqueous and oil) are then mixed by repetitively pushing the plungers until a homogeneous emulsion with dispersed oil droplets is obtained. Image created with Biorender. **b**, Representative fluorescence microscopy pictures of MOE and SOE. Oil drops are stained with Nile Red (red) and proteins in the milk or syrup are stained with Fast Green (Cyan). Magnification 20x. White scale bar=20 µm. **c**, 75 µl of MOE (blue) or SOE (orange) emulsions were pipetted using the reverse pipetting approach and weighed (n=20). Depicted is the % of variation in pipetting (relative to the average weight) ±SD for each formulation.

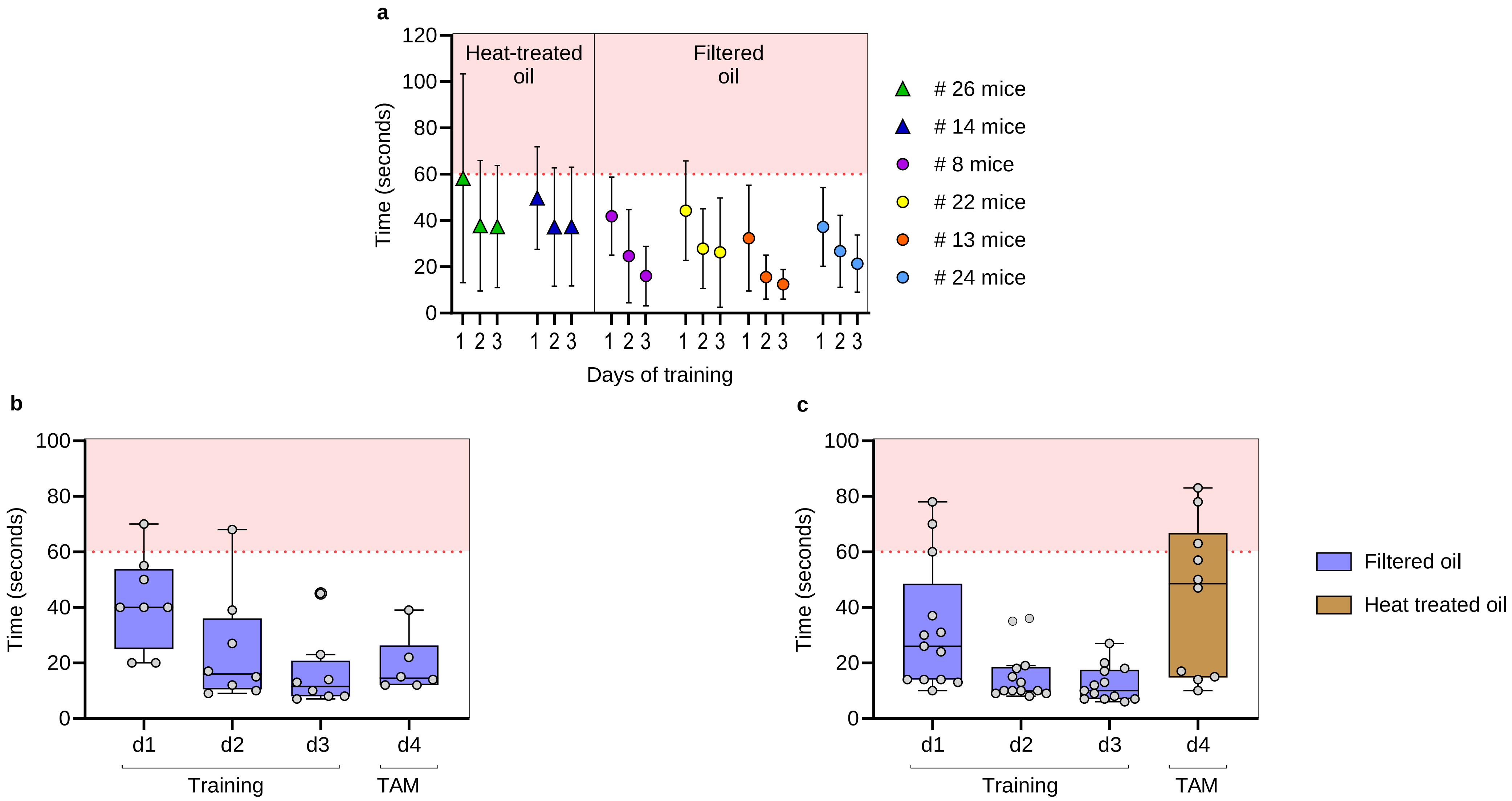

Figure S2: Palpability of emulsions made with filtered or heat-treated oil. a, Compilation of consumption times for three days of training, recorded and depicted as described in Figure 1e. Data is from different experiments where adult male and female mice were offered sweetened milk-oil emulsions (MOE) made with either heat-treated oil (triangles) or sterile filtered oil (circles). Each experiment is indicated with a different color and the number of mice per experiment is indicated in the legend. The mean consumption time ± SD is indicated for each training day. **b** and **c**, Consumption times during training with MOE made with filtered oil (blue bars) and MOE-TAM treatment made with emulsions containing filtered oil (**b**) or heat-treated oil (**c**). Tukey box plots in graphs show the minimum and maximum values (ends of the whiskers), interquartile range (length of the box), and median (line through the box) of sets of data. Individual data points are shown as circles.

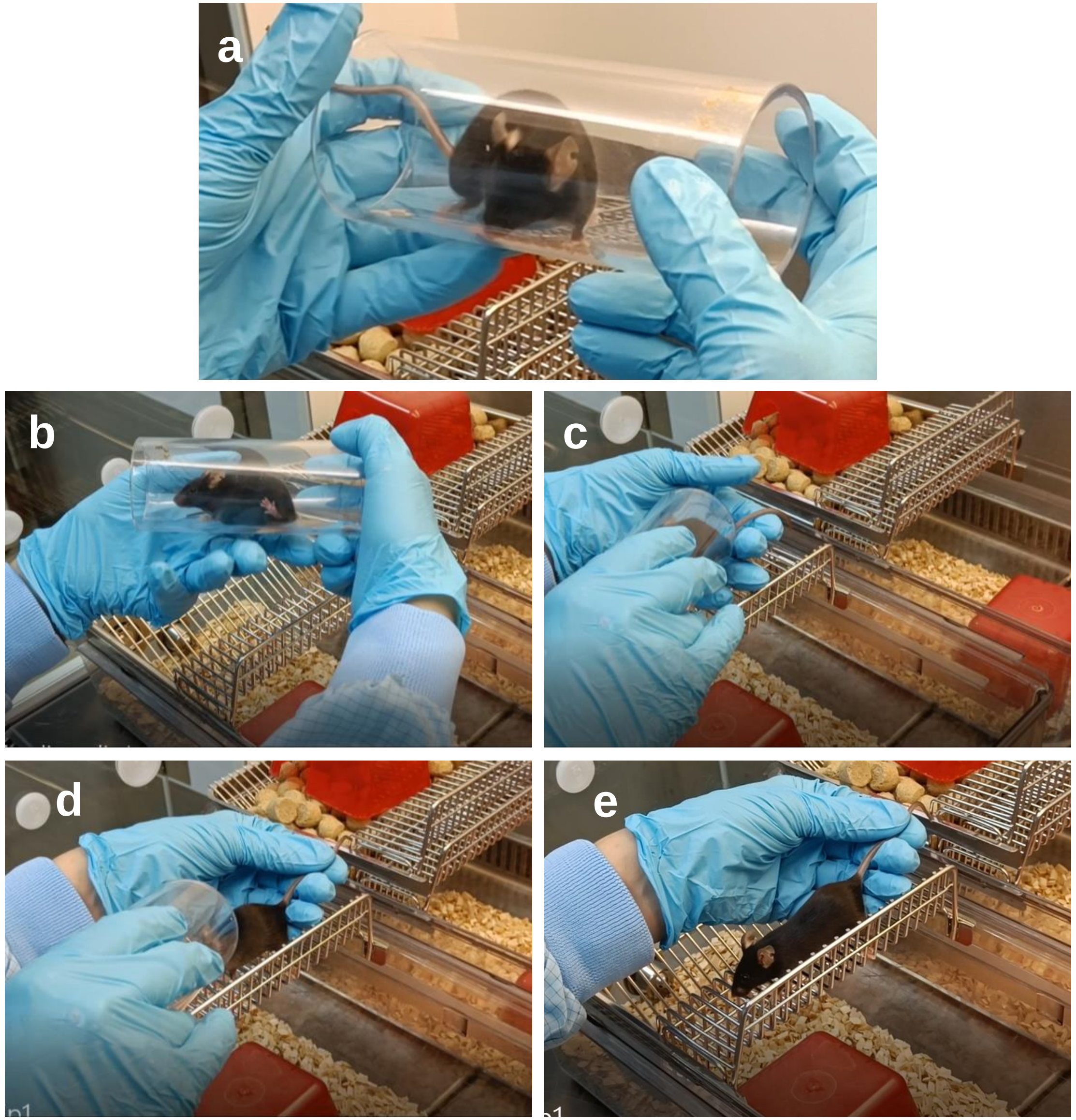

Figure S3: Handling of mice using training tunnels. Mice were habituated to tunnels for at least two days prior to training and treatments. **a**, Mice were collected from the home cage using the tunnel and identified based on earmarks. **b-e**, The animals were transferred to the grid of an empty cage and offered a formulation with a micropipette while gently held by the proximal part of the tail (see also Figure 1b).

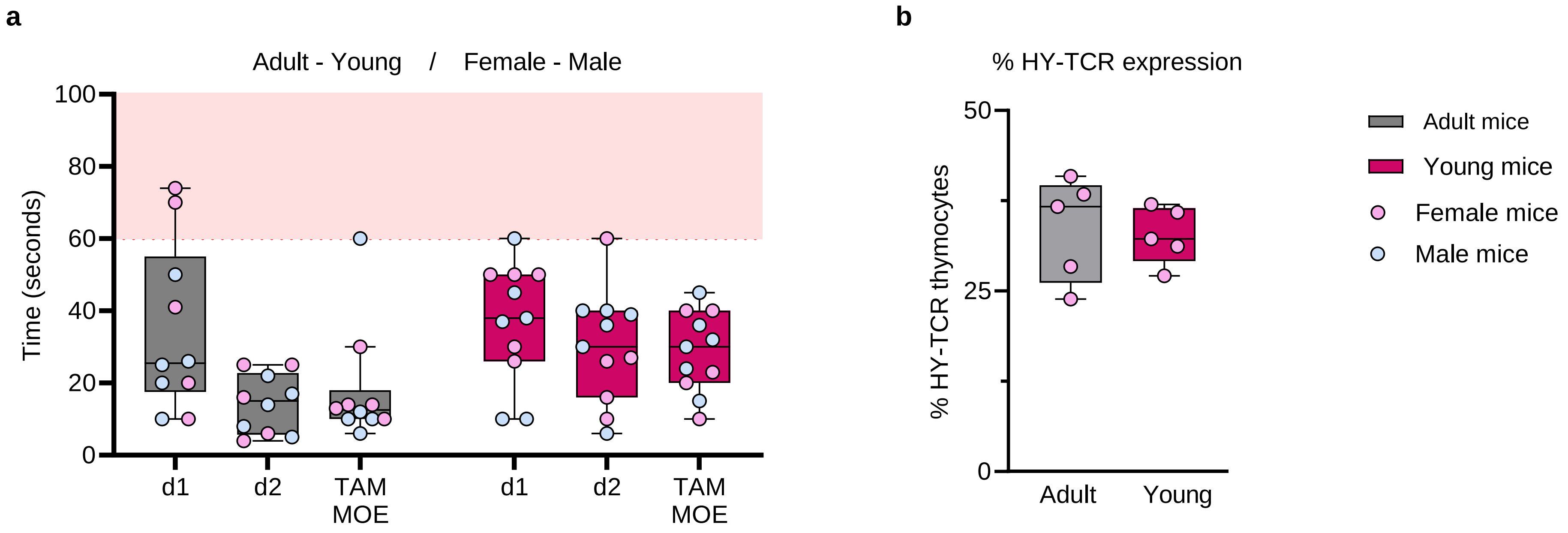

Figure S4: Pipette feeding of young or adult, male and female mice. Adult (13 weeks) and pre-puberty (5 weeks) male and female mice (n=5 per group) were trained for 2 days and pipette-fed with 40 mg/kg MOE-TAM on day 3. **a**, Consumption time during training with MOE and treatment with MOE-TAM was recorded and depicted as described in Figure 1e. **b**, Comparison of CreER^{T2}-dependent HY-TCR reporter expression in thymocytes of female young and adult mice shown in (**a**), 40 hours after treatment with a single dose of 40 mg/kg TAM. Only HY-TCR induction results of female mice are shown since in males HY-TCR thymocytes are autoreactive and eliminated due to negative selection events. The results are shown as Tukey boxplots, including minimum and maximum values, interquartile range and median with individual data points overlayed as scatter dot plots.

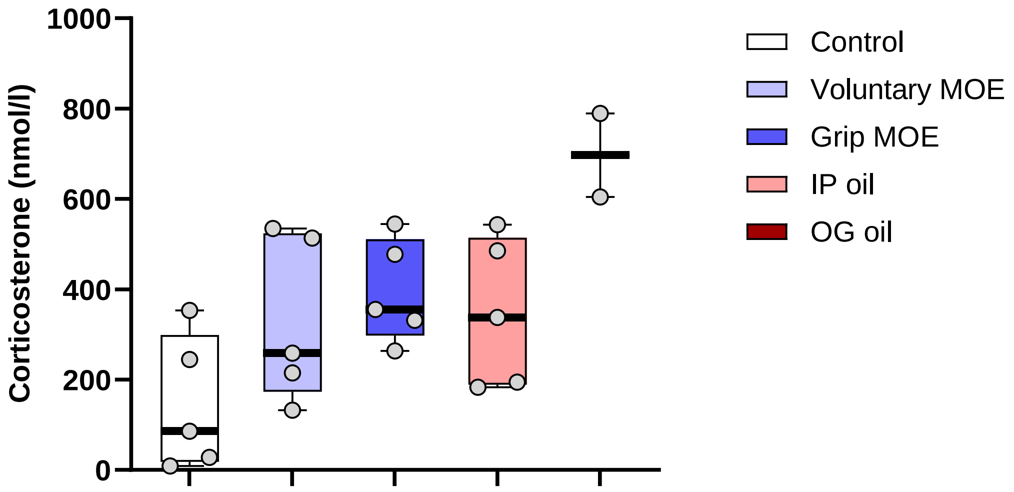

Fig. S5: Effects of administration methods on acute stress response. Plasma corticosterone levels were collected 30 min after micropipette-feeding the mice with MOE while on the grid (Voluntary MOE, n=5) or while gently held at the scruff (Grip MOE, n=5) or after mice were treated with oil via intraperitoneal (IP oil, n=5) injections or oral gavage (OG oil, n=2). Both pipette-fed groups were trained for 2 days prior to feeding and testing for CORT plasma levels. Non-treated mice (Control) were used as a negative control. Results are from male and female adult mice.

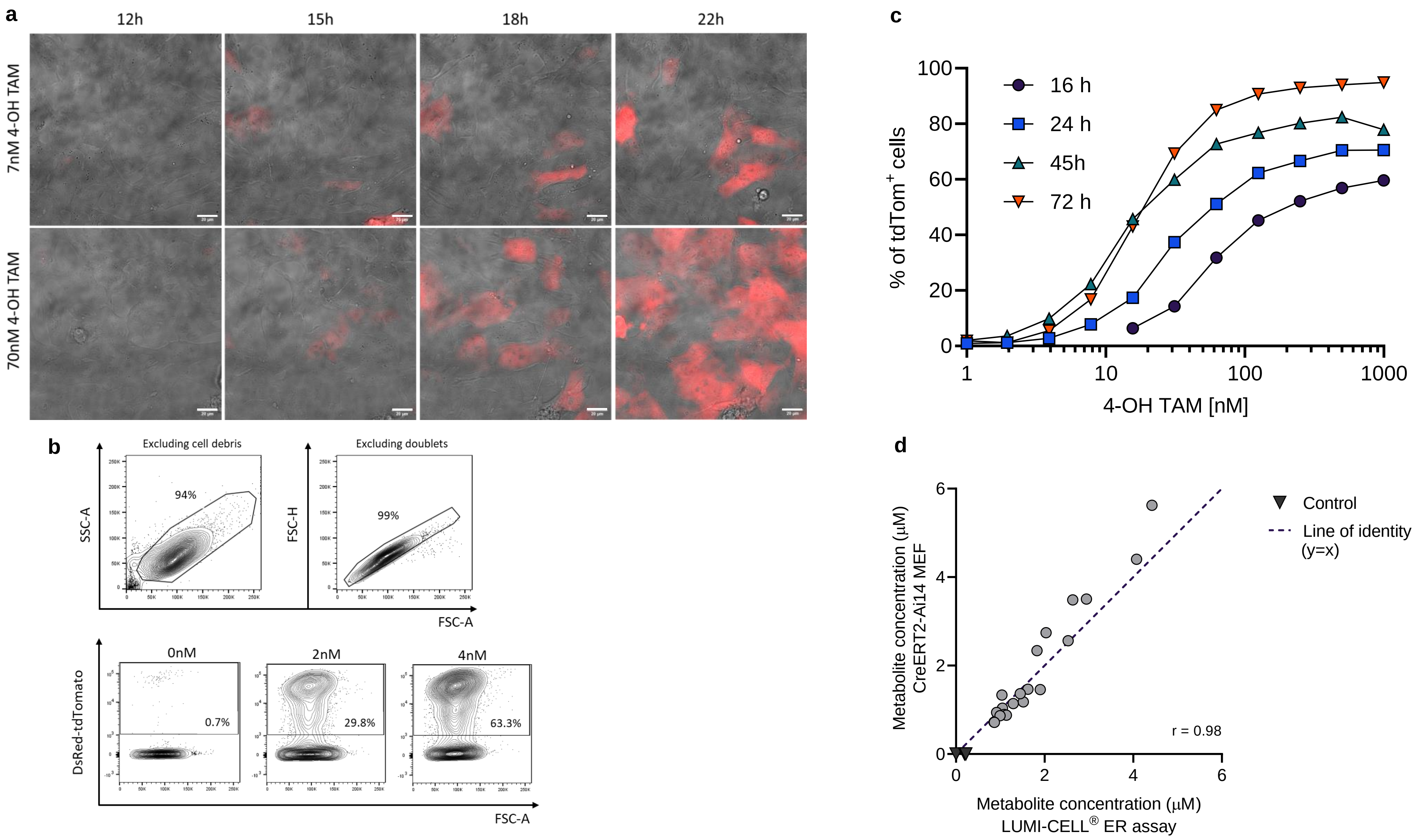

Figure S6: Specifics of an in vitro assay for the quantitative detection of serum TAM metabolites. a, R26-Cre-ER^{T2} Ai14 mesenchymal embryonic fibroblast cells (MEF) were cultured and treated with 7 nM (top) or 70 nM (bottom panels) 4-OH TAM for 28 hours. Induction of tdTomato expression (red) was followed over time (bright field and fluorescence microscopy) at indicated time points with magnification 63x. Scale bar = 50 µm. **b**, Representative dot plots of a flow cytometric analysis of R26-Cre-ERT2 Ai14 MEFs treated with 0 nM, 2 nM or 4 nM OH-TAM for 45 hours. Gates on the dot plots were set to exclude cell debris and doublets (top panels) and to calculate the percentage tdTomato expressing MEFs (bottom panels). **c**, Percentage of tdTomato expressing cells, analyzed as shown in (**b**), following stimulation with a range of 4-OH TAM concentrations (1-1000 nM, 2-fold dilution) for the indicated time periods (1-72 h). Each data point is the average of independent duplicate measurements. **d**, TAM-metabolite concentrations in sera from mice collected 6 h after TAM treatment, were determined using R26-Cre-ERT2 Ai14 MEFs and compared to that obtained using an independent LUMI-CELL ER assay. MEF cells were cultured in the presence of the different mouse sera for 45 hours and the percentage of tdTomato positive cells quantified as shown in (**b**). The concentration was calculated based the response of the cells to a range of 4-OH TAM concentrations, resulting in a standard curve similar as shown in (**c**). Results obtained with both R26-Cre-ER^{T2} Ai14 MEFs and the LUMI-CELL ER assay were compared for each serum sample. A line of identity (y=x) was inserted as a reference for similarity and the correlation coefficient r was calculated.

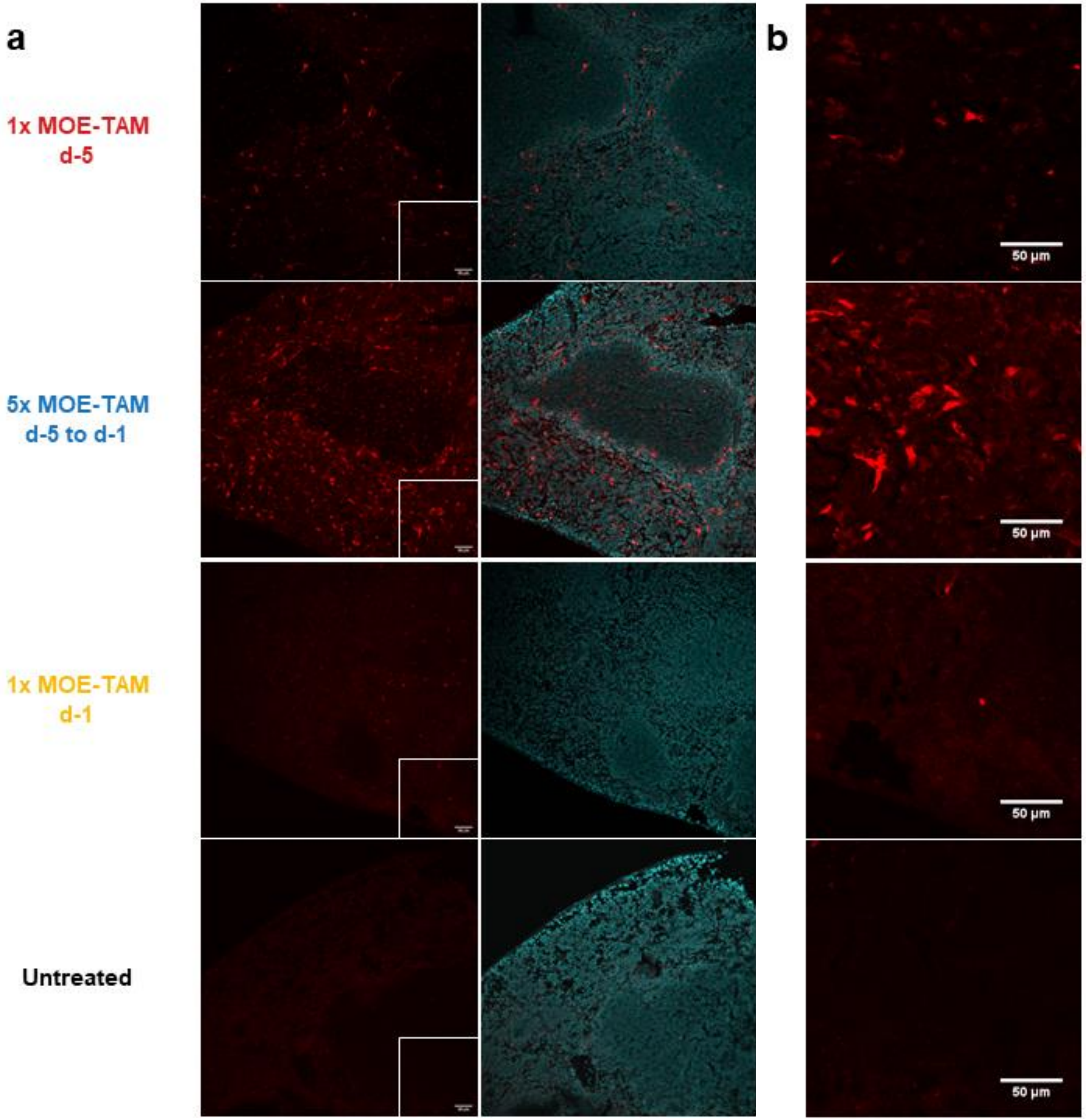

Figure S7: Repeated administration of MOE-TAM increases the induction of reporter gene expression. a, R26-Cre-ER^{T2} Ai14 adult, male or female mice were treated as specified in Figure 4a with either 1x TAM on d-5 or d-1 or 5 daily treatments on consecutive days (d-5 to d-1). Induction of tdTomato expression (red) was determined in spleen sections that were counterstained with DAPI (Cyan) and using fluorescence microscopy with 20x magnification. Scale bar = 50 µm. Representative images are shown for each group. (A) The left panels show only tdTomato fluorescence, and the right panels show a composite of tdTomato and DAPI fluorescence of the same sample. All images were processed using identical settings for each channel. **b**, Magnified view of area indicated with a white rectangle in panels in (**a**).

Product	Syrup	Oil	Sweetened milk
**Amount**	**100 g**	**100 g**	**100 g**
**Energy (kJ)**	1.048	3.700	1.356

Product (method)	Oil (IP)	Oil (OG)	MOE (Pipette)	SOE (Pipette)
**Amount**	**100 µl**	**200 µl**	**80 µl**	**80 µl**
**Energy (kJ)**	3,3	6,6	1,4	1,6
**% sugar**	0	0	9	54

Table S1: Energy content of solutions and formulations. The energy content (kJ) for 100 g of the original syrup and sweetened milk solutions was obtained from the respective product descriptions, that of peanut oil from the Swiss Food Composition Database maintained by the Federal Food Safety and Veterinary Office FSVO (https://naehrwertdaten.ch/en/search/#/food/340003).

The energy content of administered Oil, MOE and SOE was calculated based on the volume (amount) administered for a mouse of 25 g body weight and the relative content of the formulations of oil and milk (MOE) or oil and syrup (SOE).

a

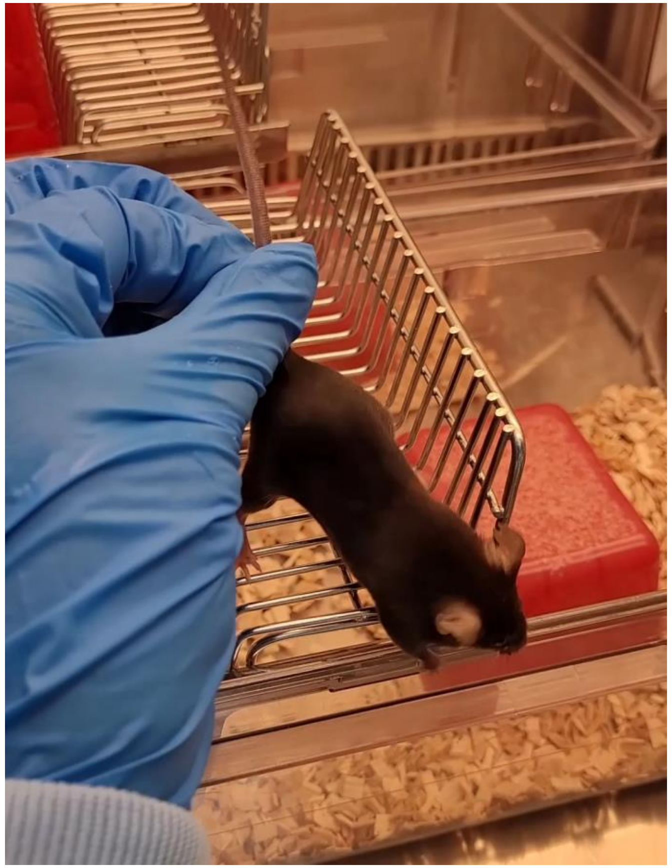

b

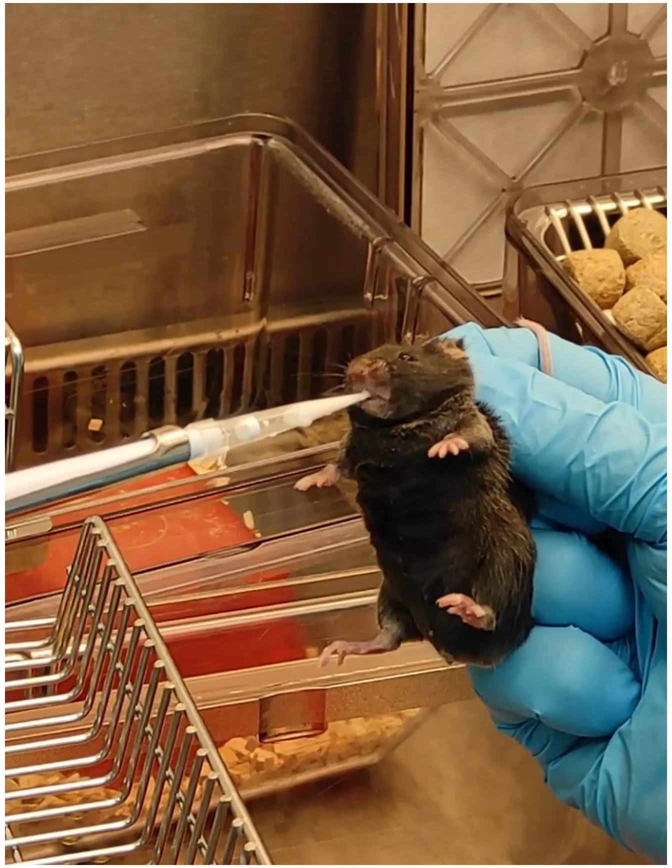

Supplementary information to Video 1 (screenshot “a”) and Video 2 (screenshot “b”), demonstrating voluntary and restrained consumption of milk-oil-emulsions. Experimental setup for training and TAM treatment of mice includes 2 new cages (left) and the home cage (right). Mice are collected from the home cage and placed on the new cage using a tunnel as shown in Figure S3. **a**, Screenshot of Video 1 showing a representative example of voluntary consumption. The mouse is gently held by the proximal tail with one hand and offered the solution using a 200 µl pipette with the other hand. Once the mouse shows interest in the tip/offered solution, small volumes are expelled according to how fast the mouse drinks the solution. **b**, Screenshot of Video 2 showing a representative example of restrained consumption. The mouse is gently restrained by holding the scruff of the neck with one hand while it is offered the formulation with the other. Similar as for voluntary consumption, small volumes are expelled from the pipette tip according to how fast the mouse drinks the solution. **a** and **b**, After the mice consume the offered solution, they are either temporarily placed in a separate cage (when multiple mice need treatment) or returned directly to the home cage.
