# Supplementary material for: Refined tamoxifen administration in mice by encouraging voluntary consumption of palatable formulations
Source: Lab Anim (NY). 2024 Jul 30;53(8):205–14. doi: 10.1038/s41684-024-01409-z (PMC11291282; doi:10.1038/s41684-024-01409-z)
Supplement: Supplementary file 2 — Reporting Summary [file 41684_2024_1409_MOESM2_ESM.pdf]

nature portfolio

Corresponding author(s): Thorsten Buch

Last updated by author(s): Jun 20, 2024

Reporting Summary

Nature Portfolio wishes to improve the reproducibility of the work that we publish. This form provides structure for consistency and transparency in reporting. For further information on Nature Portfolio policies, see our Editorial Policies and the Editorial Policy Checklist.

Statistics

For all statistical analyses, confirm that the following items are present in the figure legend, table legend, main text, or Methods section.

n/a	Confirmed	
☐	☒	The exact sample size (*n*) for each experimental group/condition, given as a discrete number and unit of measurement
☐	☒	A statement on whether measurements were taken from distinct samples or whether the same sample was measured repeatedly
☐	☒	The statistical test(s) used AND whether they are one- or two-sided<br>*Only common tests should be described solely by name; describe more complex techniques in the Methods section.*
☒	☐	A description of all covariates tested
☐	☒	A description of any assumptions or corrections, such as tests of normality and adjustment for multiple comparisons
☐	☒	A full description of the statistical parameters including central tendency (e.g. means) or other basic estimates (e.g. regression coefficient) AND variation (e.g. standard deviation) or associated estimates of uncertainty (e.g. confidence intervals)
☐	☒	For null hypothesis testing, the test statistic (e.g. *F*, *t*, *r*) with confidence intervals, effect sizes, degrees of freedom and *P* value noted<br>*Give P values as exact values whenever suitable.*
☒	☐	For Bayesian analysis, information on the choice of priors and Markov chain Monte Carlo settings
☒	☐	For hierarchical and complex designs, identification of the appropriate level for tests and full reporting of outcomes
☐	☒	Estimates of effect sizes (e.g. Cohen's *d*, Pearson's *r*), indicating how they were calculated

Our web collection on statistics for biologists contains articles on many of the points above.

Software and code

Policy information about availability of computer code

Data collection	LSRII Fortessa,(Becton Dickinson), TECAN reader (SPARK®TECAN), Zeiss, Axio Observer Z1, Leica DMI6000 AFC Model SP8, Aurora (Cytek) spectral flow analyzer
Data analysis	FlowJo 10.4, Prism v9.2.0, GraphPad, ImageJ (v1.53c), QuPath v0.3.0, Excel MS 365 Microsoft.

For manuscripts utilizing custom algorithms or software that are central to the research but not yet described in published literature, software must be made available to editors and reviewers. We strongly encourage code deposition in a community repository (e.g. GitHub). See the Nature Portfolio guidelines for submitting code & software for further information.

Data

Policy information about availability of data

All manuscripts must include a data availability statement. This statement should provide the following information, where applicable:

- Accession codes, unique identifiers, or web links for publicly available datasets
- A description of any restrictions on data availability
- For clinical datasets or third party data, please ensure that the statement adheres to our policy

The data underlying examples and figures are available from the corresponding author or from publicly available repository (https://doi.org/10.5281/zenodo.11978858 (2024).)

Field-specific reporting

Please select the one below that is the best fit for your research. If you are not sure, read the appropriate sections before making your selection.

☒ Life sciences ☐ Behavioural & social sciences ☐ Ecological, evolutionary & environmental sciences

For a reference copy of the document with all sections, see nature.com/documents/nr-reporting-summary-flat.pdf

Life sciences study design

All studies must disclose on these points even when the disclosure is negative.

Sample size	The sample size for each experiment is indicated in the text or the figure legends. Power calculations to determine the group size for the non-inferiority test were performed in the statistical environment R, using a one-tailed t-test with a power of 0.8 and a significance level (alpha) of 0.025 or 0.05 as indicated in the text.
Data exclusions	No data were excluded.
Replication	At least three replicates were analyzed in at least two independent experiments. The obtained experimental results were reliably reproduced.
Randomization	The animals were randomly allocated to groups. Groups with different treatments were kept in separate cages.
Blinding	In vitro assays with mouse sera were performed with the experimenter blinded to the origin of the samples. Treatment of mice were blinded to the experimenter when possible.

Reporting for specific materials, systems and methods

We require information from authors about some types of materials, experimental systems and methods used in many studies. Here, indicate whether each material, system or method listed is relevant to your study. If you are not sure if a list item applies to your research, read the appropriate section before selecting a response.

Materials & experimental systems

n/a	Involved in the study	
☐	☒	Antibodies
☐	☒	Eukaryotic cell lines
☒	☐	Palaeontology and archaeology
☐	☒	Animals and other organisms
☒	☐	Human research participants
☒	☐	Clinical data
☒	☐	Dual use research of concern

Methods

n/a	Involved in the study	
☒	☐	ChIP-seq
☐	☒	Flow cytometry
☒	☐	MRI-based neuroimaging

Antibodies

Antibodies used	AQUA Zombie live/dead dye (BioLegend #423101)<br>Zombie NIR live/dead dye (Biolegend #423105)<br>Anti-TCR β (BioLegend #2629564)<br>Anti-CD8a (BioLegend #2562610)<br>Anti-CD4 (BioLegend #893330)<br>Anti-TCR H-Y (eBioScience #466267)<br>Anti-CD3e (eBioScience #469315)<br>Anti-NK1.1 (BioLegend #313312)<br>Anti-CD19 (BioLegend #313641)<br>Anti-CD25 (BioLegend #313392)<br>Anti-Fc receptor blocking antibody (BioLegend AB_1574973)<br>Anti-CD4 (Biolegend #100428)
Validation	Only commercially available antibodies were used and these were validated by the manufacturer or in previous publications.

Eukaryotic cell lines

Policy information about cell lines

Cell line source(s)	VM7Luc4E2 cell line was generated by Prof. M. S. Denison (University of California – Davis) by transfection of human MCF7 breast cancer cells with the pGudLuc plasmid.<br>R26-Cre-ERT2/Ai14 MEFs: Embryonic fibroblasts: Mesenchymal embryonic fibroblasts (MEF) isolated from individual R26-CreERT2-Ai14 mice.
Authentication	The VM7Luc4E2 cell line was authenticated as published by Prof Denison. The MEF cell lines were genotyped for the presence of both transgenes.
Mycoplasma contamination	All cell lines tested negative for mycoplasma (based on PCR determination)
Commonly misidentified lines (See ICLAC register)	Not applicable

Animals and other organisms

Policy information about studies involving animals; ARRIVE guidelines recommended for reporting animal research

Laboratory animals	Both male and female mice were used and analyzed at age 10-18 weeks old, except for HY-switch model where only female mice were used to determine induction of the transgenic HY-TCR gene.
Wild animals	The study did not involve wild animals.
Field-collected samples	No field collected samples were used in this study.
Ethics oversight	All experimental procedures described were approved by the Cantonal Veterinarian’s Office of Zürich.

Note that full information on the approval of the study protocol must also be provided in the manuscript.

Flow Cytometry

Plots

Confirm that:

☒ The axis labels state the marker and fluorochrome used (e.g. CD4-FITC).

☒ The axis scales are clearly visible. Include numbers along axes only for bottom left plot of group (a 'group' is an analysis of identical markers).

☒ All plots are contour plots with outliers or pseudocolor plots.

☒ A numerical value for number of cells or percentage (with statistics) is provided.

Methodology

Sample preparation	Single cell suspension were prepared in cold DPBS supplemented with 2% FCS and 0.05% NaN3.
Instrument	LSRII Fortessa, (Becton Dickinson), Aurora (Cytek) spectral flow analyzer
Software	FlowJo 10.4
Cell population abundance	Not applicable
Gating strategy	Live cells were defined by FSC gating and staining with LIVE/DEAD Fixable Aqua Dead Cell Stain Kit. All gating strategies are stated in the manuscript.

☒ Tick this box to confirm that a figure exemplifying the gating strategy is provided in the Supplementary Information.
